# Angiogenesis-related proteins as biomarkers for peripheral artery disease

**DOI:** 10.1016/j.heliyon.2023.e20166

**Published:** 2023-09-15

**Authors:** Ben Li, Niousha Djahanpour, Abdelrahman Zamzam, Muzammil H. Syed, Shubha Jain, Rawand Abdin, Mohammad Qadura

**Affiliations:** a Division of Vascular Surgery, St. Michael's Hospital, Unity Health Toronto, University of Toronto, Canada; b Department of Medicine, McMaster University, Hamilton, Canada; c Department of Surgery, University of Toronto, Toronto, Canada; d Keenan Research Centre for Biomedical Science, Li Ka Shing Knowledge Institute, St. Michael's Hospital, Unity Health Toronto, University of Toronto, Canada

**Keywords:** Angiogenesis-related proteins, Hepatocyte growth factor (HGF), Biomarkers, Peripheral artery disease

## Abstract

**Background:**

Angiogenesis plays an important role in peripheral artery disease (PAD) and angiogenesis-related proteins may act as prognostic biomarkers. This study assesses the potential for angiogenesis-related proteins to predict adverse events associated with PAD.

**Methods:**

This was a case-control study. Patients with PAD (n = 250) and without PAD (n = 125) provided blood samples and were followed prospectively for three years. Concentrations of 17 angiogenesis-related proteins were measured in plasma. The incidence of major adverse limb event (MALE), defined as a composite of major amputation or vascular intervention, was the primary outcome. Worsening PAD status, defined as a drop in ankle brachial index ≥ 0.15, was the secondary outcome. Multivariable regression adjusted for baseline characteristics was conducted to determine the prognostication value of angiogenesis-related proteins in predicting MALE.

**Findings:**

Relative to patients without PAD, 8 proteins related to angiogenesis were expressed differentially in PAD patients. Worsening PAD status and MALE were observed in 52 (14%) and 83 (22%) patients, respectively. Hepatocyte growth factor (HGF) was the most reliable predictor of MALE (adjusted HR 0.79, 95% CI 0.15–0.86). Compared to individuals with high HGF, patients with low HGF had a decreased three-year freedom from MALE [66% vs 88%, p = 0.001], major amputation [93% vs 98%, p = 0.023], vascular intervention [68% vs 88%, p = 0.001], and worsening PAD status [81% vs 91%, p = 0.006].

**Interpretation:**

Measuring plasma levels of HGF in individuals with PAD can assist in identifying patients at elevated risk of adverse events related to PAD who may benefit from additional evaluation or treatment.

## 1 Introduction

Angiogenesis is defined as the development of new blood vessels, which plays an important role in cardiovascular disease (CVD) [1]. The vascular system consists of arteries, veins, and capillaries that provide oxygen and nutrients to vital organs [2]. Endothelial cells (EC's) line blood vessels and regulate the communication between the vasculature and surrounding environment [3]. Physiologically, EC's remain in a quiescent state and rarely proliferate; however, they can initiate angiogenesis in ischemic/hypoxic environments [4]. Atherosclerosis is a progressive disease characterized by fibrofatty lesion formation on arterial walls leading to downstream ischemia [5]. Given that ischemia induced by atherosclerosis may trigger angiogenesis, the intricate relationship between these two entities is important in understanding CVD development [6]. The clinical importance of angiogenesis has been studied in several CVD's, including cerebrovascular disease, coronary artery disease, and peripheral artery disease [7].

Peripheral artery disease (PAD) is a chronic atherosclerotic disorder resulting in lower limb ischemia [8]. This condition affects over 150 million people globally and although it is strongly correlated with amputation and mortality, PAD is poorly diagnosed and treated [9]. The ankle brachial index (ABI) remains the most widely used instrument for PAD screening; however it has multiple limitations, including inaccuracy in diabetics and dependence on the operator [10,11]. Furthermore, the ABI is a limited prognostic tool as it is a relatively poor predictor of outcomes in patients with PAD [12]. Identifying improved markers for PAD prognostication could strengthen stratification of patients based on risk of complications. This would in turn allow clinicians to better identify patients for additional work-up and therapy [13]. Furthermore, high risk patients may benefit from early surgery to increase blood flow towards the lower extremities to prevent limb loss [14].

Several angiogenesis factors have been demonstrated to be associated with PAD, including vascular endothelial growth factor (VEGF), fibroblast growth factor (FGF), and hepatocyte growth factor (HGF)and [15]. Administration of these factors intramuscularly or intra-arterially in animal models can stimulate angiogenesis [15]. Since angiogenesis is important in supporting blood flow to ischemic tissue, our hypothesis was that altered blood concentrations of these angiogenesis-related proteins could be correlated with complicated related to PAD, thereby acting as biomarkers for PAD prognosis. This study assessed the prognostic potential of various angiogenesis-related proteins in patients with PAD.

## 2 Methods

### 2.1 Ethical approval

The Unity Health Toronto research ethics board approved this study. All patients provided informed consent and the study was performed according to principles outlined in the Declaration of Helsinki [16].

### 2.2 Patient recruitment

Consecutive patients with and without PAD attending vascular clinics at our institution between January–December 2018 were recruited. PAD was defined as per medical guidelines (ABI below 0.9 or toe brachial index (TBI) below 0.7, with or without claudication and absent or decreased pedal pulses) [17]. We included patients with the following Fontaine stages: I [asymptomatic], IIa [claudication at greater than 200 m], IIb [claudication at less than 200 m], and III [chronic limb threatening ischemia: rest pain]. There were no patients at stage IV (necrosis or gangrene) because they were excluded due to requiring vascular intervention or amputation at baseline. Fontaine classification was defined based on the published literature [18]. Patients with one or more of these conditions were excluded: stage 3–5 chronic kidney disease (defined as estimated glomerular filtration rate below 60 mL/min/1.73 m^2^), acute coronary syndrome, or acute limb ischemia within 3 months prior to enrollment.

### 2.3 Baseline characteristics

Documented baseline characteristics included age, biological sex, hypertension, dyslipidemia, diabetes, smoking status, congestive heart failure, coronary artery disease, and ABI [19]. Cardiovascular risk factors were defined using the guidelines provided by the American College of Cardiology [20,21].

### 2.4 Angiogenesis factors

Several angiogenesis factors were analyzed: endothelial growth factor (EGF), angiopoietin-2, endothelin-1, interleukin-8 (IL-8), vascular endothelial growth factors (VEGF) A, C, and D, fibroblast growth factor (FGF) 1 and 2, granulocyte colony stimulating factor (G-CSF), leptin, bone morphogenic protein 9 (BMP-9), hepatocyte growth factor (HGF), endoglin, follistatin, heparin-binding epidermal growth factor-like growth factor (HB-EGF), and placental growth factor (PLGF). These factors were selected given their previously demonstrated correlations with PAD [[22], [23], [24], [25]].

### 2.5 Measurement of protein concentrations

Patients provided plasma samples and angiogenesis factor levels were analyzed using a magnetic bead panel specific to human cardiovascular diseases (EMD-Millipore; Billerica, MA) [26]. The MagPix analyzer was calibrated using Luminex Corp bead kits (Luminex Corp; Austin, Texas) [27]. A minimum of fifty beads for each angiogenesis-related protein were obtained using xPonent software (Luminex) and assessed using Milliplex tool (v5.1; EMD-Millipore) [28].

### 2.6 Outcomes

Three-year major adverse limb events (MALE) [composite of major amputation (above the ankle) or vascular intervention (open/endovascular revascularization of the lower extremity) was the primary outcome. Three-year worsening PAD status (ABI drop ≥0.15) was the secondary outcome [[29], [30], [31]].

### 2.7 Statistical analysis

Means (SD) and numbers (%) were used to summarize covariates and outcomes. Between-group differences were assessed with independent *t*-test [continuous variables] and chi-square test [categorical variables]. Cox proportional hazards analysis was conducted to assess the independent association between angiogenesis-related proteins and MALE, adjusting for all recorded baseline variables. Then, the sample was split into individuals with low vs. high HGF levels, the angiogenesis factor that predicted MALE most reliably, using the median plasma concentration in our sample of 0.275 μg/mL. Kaplan-Meier curves were used to summarize event-free survival rates of both groups, and between-group differences were assessed using log-rank test. Stratified analysis was performed based on Fontaine classification to assess the association between outcomes and PAD severity. Between-group differences were determined by performing one-way analysis of variance (ANOVA). The coefficient of determination (R^2^) was calculated to determine the strength of association between HGF levels and Fontaine classification. A two-tailed p < 0.05 was used as the threshold of statistical significance. SPSS version 23 (SPSS Inc., Chicago, Illinois, USA) was used to perform all analyses [32].

### 2.8 Role of funders

Funders did not play a role in designing or conducting this research.

## 3 Results

### 3.1 Patient characteristics: patients with PAD were more commonly diagnosed with traditional cardiac risk factors

Overall, 375 consenting patients were enrolled, of whom 250 had PAD while the remaining 125 were non-PAD. Age and sex did not differ among both patient groups. Compared to the non-PAD group, a larger proportion of PAD patients had comorbidities and risk factors commonly associated with CVD's including diabetes (42% vs 18%, p = 0.001), previous stroke (21% vs 10%, p = 0.01), congestive heart failure (6% vs 0%, p = 0.007), and current smoking status (30% vs 19%, p = 0.014) (Table 1).

**Table 1:** Baseline traditional cardiovascular risk factors in cohort.

	Overall (n = 375)	Non-PAD (n = 125)	PAD (n = 250)	*P*-value
**Mean (SD)**				
Age	68 (10)	67 (11)	69 (9)	0.213
**N (%)**				
Sex, male	260 (69)	90 (72)	170 (68)	0.514
Hypertension	278 (74)	85 (68)	193 (77)	0.055
Dyslipidemia	300 (80)	96 (77)	204 (82)	0.273
Diabetes	127 (34)	23 (18)	104 (42)	**0.001**
Chronic kidney disease	11 (3)	2 (2)	9 (4)	0.224
Past smoker	196 (52)	65 (52)	131 (53)	0.942
Current smoker	99 (26)	24 (19)	75 (30)	**0.014**
Congestive heart failure	14 (4)	0 (0)	14 (6)	**0.007**
Coronary artery disease	150 (40)	45 (36)	105 (42)	0.264
Previous stroke	66 (18)	13 (10)	53 (21)	**0.010**

Abbreviations: PAD (peripheral artery disease), SD (standard deviation).

### 3.2 Angiogenesis-related proteins levels are different among patients with and without PAD

Baseline levels of angiogenesis-related proteins were compared between patients with PAD and those without PAD. In comparison to individuals without PAD, PAD patients demonstrated higher mean [SD] plasma concentrations of FGF-1 (12.51 [8.41] vs 9.76 [4.44] pg/mL, p = 0.031) and leptin (30.6 [10.3] vs 22.9 [14.2] ug/mL, p = 0.035). In comparison to individuals without PAD, PAD patients demonstrated lower mean [SD] plasma concentrations of HGF (0.20 [0.05] vs 0.33 [0.23] ug/mL, p = 0.011], BMP-9 (0.13 [0.17] vs 0.18 [0.02] ug/mL, p = 0.022), endoglin (1.32 [0.23] vs 1.54 [0.16] ug/mL, p = 0.01), follistatin (0.52 [0.32] vs. 0.76 [0.38] ug/mL, p = 0.003), HB-EGF (0.02 [0.01] vs 0.05 [0.04] ug/mL, p = 0.044), and PLGF (3.01 [3.17] vs. 5.04 [2.92] pg/mL, p = 0.002). Our analysis demonstrated no significant differences in EGF, angiopoietin-2, endothelin-1, IL-8, VEGF-A, C, and D, FGF-2, and G-CSF between patients with and without PAD (Table 2).

**Table 2:** Baseline levels of angiogenesis-related proteins in cohort.

	Non-PAD (n = 125)	PAD (n = 250)	*P*-value
EGF (pg/ml)	59.3 (9.12)	65.3 (10.4)	0.586
Angiopoietin-2 (ug/ml	2.05 (1.43)	1.99 (1.31)	0.704
Endothelin-1 (pg/ml)	5.88 (3.16)	4.47 (2.78)	0.094
IL-8 (pg/ml)	6.92 (4.51)	6.57 (3.09)	0.596
VEGF-C (ug/ml)	0.97 (0.60)	0.98 (0.64)	0.913
FGF-2 (ug/ml)	0.12 (0.08)	0.11 (0.09)	0.145
VEGF-A (ug/ml)	0.12 (0.10)	0.13 (0.11)	0.750
FGF-1 (pg/ml)	9.76 (4.44)	12.51 (8.41)	**0.031**
G-CSF (pg/ml)	0.10 (0.01)	0.14 (0.03)	0.057
Leptin (ug/ml)	22.9 (14.2)	30.6 (10.3)	**0.035**
HGF (ug/ml)	0.33 (0.23)	0.20 (0.05)	**0.011**
BMP-9 (ug/ml)	0.18 (0.02)	0.13 (0.17)	**0.022**
Endoglin (ug/ml)	1.54 (0.16)	1.32 (0.23)	**0.010**
Follistatin (ug/ml)	0.76 (0.38)	0.52 (0.32)	**0.003**
HB-EGF (ug/ml)	0.05 (0.04)	0.02 (0.01)	**0.044**
PLGF (pg/ml)	5.04 (2.92)	3.01 (3.17)	**0.002**
VEGF-D (ug/ml)	0.35 (0.69)	0.33 (0.17)	0.208

Results presented as mean (standard deviation).

Abbreviations: PAD (peripheral artery disease), endothelial growth factor (EGF), interleukin-8 (IL-8), vascular endothelial growth factor (VEGF), fibroblast growth factor (FGF), granulocyte colony stimulating factor (G-CSF), leptin, bone morphogenic protein 9 (BMP-9), hepatocyte growth factor (HGF), endoglin, follistatin, placental growth factor (PLGF), heparin-binding epidermal growth factor-like growth factor (HB-EGF).

### 3.3 Outcome rates

The median follow-up time was 3 years. We noted that MALE and individual MALE outcomes occurred more frequently in PAD patients. Over 3 years, our data demonstrated that 83 (22%) patients developed MALE, all of which happened in the PAD cohort (34%). A greater proportion of PAD patients developed worsening PAD status with an ABI drop >0.15 (21% vs. 0%, p = 0.001), required major amputation (7% vs. 0%, p = 0.002), underwent vascular intervention (33% vs. 0%), and developed MALE (34% vs. 0%, p = 0.001) (Table 3).

**Table 3:** Event rates at 3 years.

	Overall (n = 375)	Non-PAD (n = 125)	PAD (n = 250)	P value
Major adverse limb event (MALE)	83 (22)	0 (0)	83 (34)	**0.001**
Vascular intervention	79 (21)	0 (0)	79 (33)	**0.001**
Major amputation	17 (5)	0 (0)	17 (7)	**0.002**
Worsening PAD status	52 (14)	0 (0)	52 (21)	**0.001**

Results presented as N (%).

Abbreviations: PAD (peripheral artery disease).

### 3.4 Some but not all investigated angiogenesis-related proteins were associated with study endpoints

**Fig. 1:**
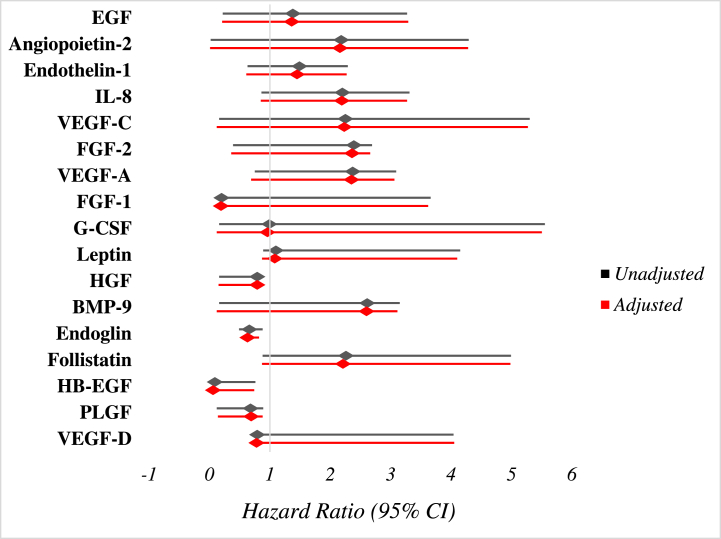
**Cox regression**analysis of the**relationship between angiogenesis-related proteins and 3-year major adverse limb events.** Multivariable models adjusted for confounding baseline variables including sex, age, diabetes, dyslipiedemia, hypertension, smoking status, chronic kidney disease, coronary artery disease, congestive heart failure and previous stroke.

Using multivariable Cox proportional hazards analysis, our data demonstrated that reduced baseline levels of the following angiogenic factors were correlated with elevated MALE risk: HGF (HR 0.79 [95% CI 0.16–0.89], adjusted HR 0.79 [95% CI 0.15–0.86]), endoglin (HR 0.66 [95% CI 0.59–0.79], adjusted HR 0.63 [95% CI 0.51–0.82]), HB-EGF (HR 0.09 [95% CI 0.03–0.76], adjusted HR 0.06 [95% CI 0.02–0.74]), and PLGF (HR 0.68 [95% CI 0.12–0.89], adjusted HR 0.69 [95% CI 0.14–0.88]) (Fig. 1). In terms of secondary outcomes, our results have demonstrated that reduced levels of HGF was the only observed predictor of worsening PAD status (HR 0.71 [95% CI 0.16–0.88], adjusted HR 0.68 [95% CI 0.15–0.86]).

### 3.5 Patient stratification based on plasma HGF concentrations

According to our analysis of primary and secondary outcomes, HGF was the only significant predictor of all PAD complications investigated. Therefore, we investigated this protein for further analysis and patient risk factor stratification. Baseline levels of HGF were used to stratify patients into low vs. high HGF using the median level of 0.275 μg/mL. This included 187 patients with low HGF and 188 patients with high HGF. Compared to individuals with high HGF, a greater proportion of patients with low HGF had PAD (76% vs 34%, p = 0.001) and diabetes (40% vs 28%, p = 0.020). Patients with low HGF had an increased incidence of 3-year MALE (34% vs 12%, p = 0.001), major amputation (7% vs 2%, p = 0.023), vascular intervention (32% vs 12%, p = 0.001), and worsening PAD status (19% vs 9%, p = 0.006) compared to patients with high HGF (Table 4).

**Table 4:** Characteristics of individuals with low vs. high hepatocyte growth factor (HGF) levels.

	Low HGF (n = 187)	High HGF (n = 188)	P value
**Mean (SD)**			
Age	68 (10)	69 (8)	0.193
**Comorbidities: N (%)**			
Peripheral artery disease	142 (76)	63 (34)	**0.001**
Sex, male	126 (67)	134 (71)	0.413
Hypertension	146 (78)	132 (70)	0.082
Dyslipidemia	153 (82)	147 (78)	0.380
Diabetes	74 (40)	53 (28)	**0.020**
Chronic kidney disease	8 (4)	3 (2)	0.124
Past smoker	97 (52)	99 (53)	0.878
Current smoker	57 (31)	42 (22)	0.093
Congestive heart failure	10 (5)	4 (2)	0.100
Coronary artery disease	75 (40)	75 (40)	0.966
Previous stroke	39 (21)	27 (14)	0.099
**Events at 3 years: N (%)**			
Major adverse limb event (MALE)	64 (34)	23 (12)	**0.001**
Vascular intervention	60 (32)	23 (12)	**0.001**
Major amputation	13 (7)	4 (2)	**0.023**
Worsening PAD status	36 (19)	17 (9)	**0.006**

Stratification of low vs high plasma HGF concentrations was determined using the median level of 0.275 μg/mL in our cohort.

Abbreviations: PAD (peripheral artery disease), SD (standard deviation).

### 3.6 Low levels of HGF are associated with reduced event-free survival

**Fig. 2:**
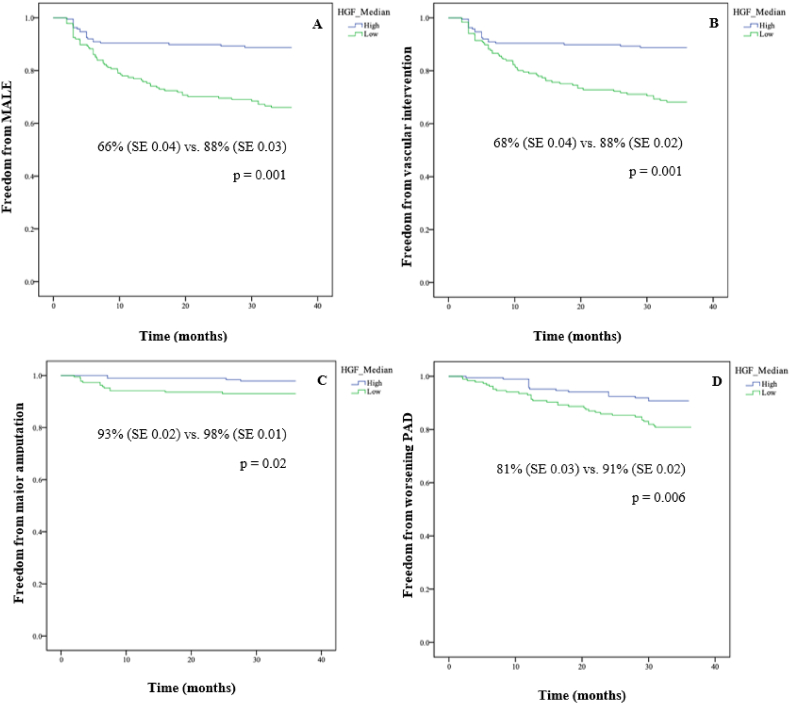
**Event-free survival in individuals with low and high hepatocyte growth factor (HGF) for A) MALE (major adverse limb event), B) vascular intervention, C) major amputation, and D) worsening PAD status.** SE (standard error). Low HGF<0.275 μg/mL (n = 187) while high HGF >0.275 μg/mL (n = 188).

**Fig. 3:**
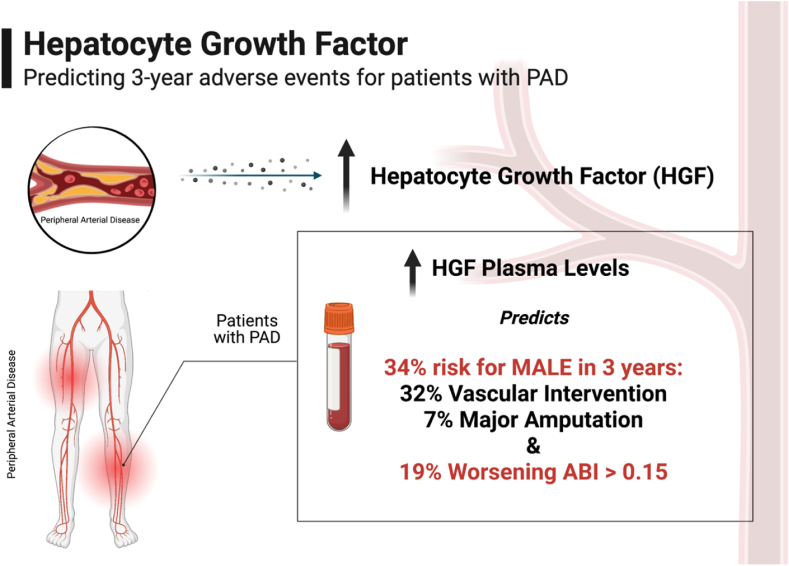
**Hepatocyte Growth Factor (HGF) as a biomarker to support prediction of peripheral artery disease (PAD) complications.** ABI (ankle brachial index), MALE (major adverse limb event). Created with permission using BioRender.com.

Patients with low plasma HGF concentrations had significantly lower rates of MALE-free survival (66% vs 88%, p = 0.001, Fig. 2A), vascular intervention-free survival (68% vs 88%, p = 0.001, Fig. 2B), amputation-free survival (93% vs 98%, p = 0.023, Fig. 2C), and worsening PAD status-free survival (81% vs 91%, p = 0.006, Fig. 2D) compared to patients with high HGF. Fig. 3 highlights the potential utility of HGF as a biomarker to predict PAD complications.

### 3.7 Stratified analysis based on fontaine classification of PAD

Patients with more severe PAD as defined by higher stages in the Fontaine classification were more likely to have 3-year MALE, major amputation, vascular intervention, and worsening PAD status (Supplemental Table 1). Furthermore, there was a significant association between lower HGF levels and higher Fontaine stages (R^2^ = 0.86) (Supplemental Fig. 1).

## 4 Discussion

### 4.1 Key findings

Herein, we demonstrated that angiogenesis-related proteins FGF-1 and leptin were elevated in patients with PAD, while HGF, endoglin, BMP-9, HB-EGF, follistatin, VEGF-D, and PLGF levels were lower in PAD patients. We further showed that HGF, endoglin, HB-EGF, and PLGF were inversely associated with MALE risk after adjusting for baseline characteristics. In addition, reduced levels of HGF were found to be associated with worsening PAD status. Upon stratification using median HGF plasma concentrations, individuals with lower HGF were more likely have PAD and develop PAD complications. Furthermore, lower HGF levels were correlated with greater PAD severity as defined by higher Fontaine stages. This investigation demonstrates the diagnostic and prognostic potential of HGF in PAD.

### 4.2 Comparison to existing literature

Guo et al. (2018) reviewed the literature on angiogenesis-related proteins in coronary artery disease and demonstrated their role in the diagnosis and management of atherosclerotic disease in coronary vessels [33]. Myocardial ischemia has been demonstrated to stimulate angiogenesis in both the acute and chronic phase within experimental models [34]. Anderson and colleagues examined the relationship between angiogenesis-related proteins and chronic kidney disease, demonstrating that VEGF-A was elevated in humans with kidney disease [35]. In animal studies, mice with induced progressive kidney disease were demonstrated to have impaired angiogenesis [36]. Süleymanoğlu et al. (2020) showed that C-reactive protein levels were associated with severity of PAD [37]. In PAD, the therapeutic potential of angiogenesis-related proteins has been studied in a Cochrane Systematic Review in 2017 [22]. The authors included 20 clinical trials studying FGF, HGF, and VEGF, and demonstrated no significant impact on major amputation or death [22]. Based on their analysis, there may be improvements in hemodynamic measures, ulceration, and rest pain [22]. On their assessment of risk-of-bias, it was concluded that the evidence quality was low due to imprecision of included studies [22]. In contrast, we did not assess the therapeutic value, but rather the prognostication potential, of proteins related to angiogenesis in PAD. We found that HGF may as act as a biomarker to help in the identification of individuals with PAD at elevated risk of adverse events.

There is some conflicting literature regarding the direction of the association between HGF levels and PAD. Garg and colleagues (2020) found that elevated HGF levels were correlated with a higher risk of PAD development [38]. In contrast, multiple investigations have shown the effectiveness of transfecting pro-angiogenic HGF plasmid DNA into patients with PAD to improve clinical outcomes [[39], [40], [41]]. The promising early results of this clinical gene therapy highlights that therapeutically increasing serum HGF levels may reduce PAD-related adverse events. Similarly, our study highlighted a strong inverse relationship between HGF levels and PAD severity based on the Fontaine classification. This study may inform a potential pathway of treatment for PAD patients. In particular, patients with decreased HGF plasma concentrations, which predicts elevated PAD disease severity, may be managed more actively with advanced drug, gene, and surgical therapy. Our study suggests a potentially different treatment approach to patients with low HGF who may be at increased risk of PAD complications. Specifically, these patients should be followed closely by a vascular specialist, receive aggressive medical management including low-dose rivaroxaban, and undergo early revascularization if needed to prevent limb loss. Further studies are required to validate our findings.

### 4.3 Explanation of findings

The first important finding of this investigation was the significant association between cardiovascular risk factors and PAD. This corroborates previous literature showing that PAD is strongly correlated with multiple comorbidities that increases the risk of adverse events [42]. Our study provides further evidence regarding the need for early diagnosis and aggressive management of PAD patients [43]. Second, we found differential expression of 8 angiogenesis-related proteins in patients with vs. without PAD. The factor with the greatest prognostic potential was HGF, a growth factor that was initially discovered as a hepatocyte mitogen responsible for liver regeneration [44]. This factor is a heterodimer molecule consisting of two subunits (69 kDa alpha and 34 kDa beta) produced by stromal cells [45]. HGF has multiple functions including morphogenesis, cellular proliferation, and angiogenesis via phosphorylation of its c-Met receptor [44]. Further investigation demonstrated that HGF produced endogenously is needed for self-repair of injured cells [44]. Relevant to cardiovascular disease, HGF has previously been demonstrated to exert potent mitogenic effects on the endothelium, thereby promoting development of collateral blood vessels, which improves tissue perfusion in patients with atherosclerotic disease [46]. Multiple investigations have demonstrated the pro-angiogenic nature of HGF, protecting patients against end-organ ischemia [[47], [48], [49]]. Hayashi and colleagues showed that hypoxia in vascular cells decreased HGF production, highlighting the critical role of HGF in the pathophysiology of ischemic conditions [50]. Similarly in our study, we found that HGF was decreased in PAD patients. Our analysis also demonstrated a strong relationship between HGF levels and severity of PAD. Although future basic science investigations are required to fully understand the mechanism for this relationship, we hypothesize that this represents impaired angiogenesis and attenuated ability to form collateral vessels in PAD patients. In turn, this may cause inadequate blood flow to the limbs, resulting in tissue ischemia. Decreased HGF plasma concentrations in individuals with severe PAD could highlight a valuable route to develop novel prognostic and therapeutic tools for the highest risk patients. Third, we found that HGF provides prognostic value in patients with PAD, as it is inversely correlated with PAD complications following adjustment for demographic and clinical variables. We found that cohort stratification into individuals with low and high HGF allowed us to identify a cohort with low HGF at elevated risk of adverse PAD complications. This demonstrates that individuals with low HGF have greater risk of developing PAD and suffer from complications of the disease. This is in line with other biomarkers including troponin and creatinine, which provide both diagnostic and prognostic value in myocardial infarction and renal failure, respectively [51,52].

### 4.4 Limitations

There are several limitations to this study. Firstly, as a prospective observational study, there existed baseline differences between groups. However, we adjusted for relevant baseline characteristics when assessing associations between plasma protein concentrations and PAD complications. Secondly, we analyzed outcomes at 3 years of follow-up. Extended follow-up may augment our ability to understand the prognostication potential value of HGF given that PAD is a chronic condition. Thirdly, individuals with specific renal and cardiovascular comorbidities were excluded to mitigate confounding risk with regards to angiogenesis-related factor levels. Hence, findings may not be applicable to all PAD patients. Additional study into HGF is necessary to better elicit how the plasma biomarker can be implemented in clinical practice.

## 5 Conclusions

As per these data, angiogenic growth factor HGF has prognostic potential in patients with PAD. Lower baseline HGF levels are correlated independently with future PAD complications. Quantifying HGF levels can strengthen identification of individuals with PAD at elevated risk of suffering major complications and could benefit from aggressive medical/surgical management. Additional investigation is required to validate our findings.

## Research in context

### Evidence before this study

There is a lack of prognostic tools for individuals suffering from peripheral artery disease (PAD), limiting effective risk stratification and management. Given the critical role of angiogenesis in PAD, we hypothesized that angiogenesis-related proteins may be correlated with adverse events related to PAD, thereby acting as prognostication biomarkers.

### Added value of this study

Our 3-year prospective case-control study assessing 17 angiogenesis-related proteins demonstrated that the most reliable major adverse limb event (MALE) predictor in PAD patients was hepatocyte growth factor (HGF). Patients with elevated levels of HGF demonstrated a significantly decreased 3-year freedom from MALE in comparison to individuals with lower concentrations of HGF (66% vs 88%).

**Implications of all the available evidence**

Measuring plasma levels of HGF in individuals with PAD can assist in identifying patients at elevated risk of complications, thereby guiding risk-stratification for additional work-up and treatment.

## Ethics statement

This study was granted approval by the Unity Health Toronto Research Ethics Board (UHT-REB). All patients provided informed consent and study procedures were conducted according to the principles outlined in the Declaration of Helsinki.

## Funding

The Blair Foundation supported this study but did not play a role in designing or conducting this research.

## Author contribution statement

Ben Li: Analyzed and interpreted the data; Wrote the paper.

Niousha Djahanpour: Performed the experiments; Analyzed and interpreted the data.

Abdelrahman Zamzam: Analyzed and interpreted the data.

Muzammil H. Syed; Shubha Jain: Performed the experiments.

Rawand Abdin; Mohammad Qadura: Conceived and designed the experiments.

## Data availability statement

Data included in article/supplementary material/referenced in article.

## Informed consent statement

All patients provided informed consent.

## Declaration of competing interest

The authors declare that they have no known competing financial interests or personal relationships that could have appeared to influence the work reported in this paper.
