# Corrigendum to “Angiogenesis-related proteins as biomarkers for peripheral artery disease” [Heliyon Volume 9, Issue 9, September 2023, Article e20166]

**DOI:** 10.1016/j.heliyon.2025.e43473

**Published:** 2025-06-06

**Authors:** Ben Li, Niousha Djahanpour, Abdelrahman Zamzam, Muzammil H. Syed, Shubha Jain, Rawand Abdin, Mohammad Qadura

**Affiliations:** a Division of Vascular Surgery, St. Michael's Hospital, Unity Health Toronto, University of Toronto, Canada; b Department of Medicine, McMaster University, Hamilton, Canada; c Department of Surgery, University of Toronto, Toronto, Canada; d Keenan Research Centre for Biomedical Science, Li Ka Shing Knowledge Institute, St. Michael's Hospital, Unity Health Toronto, University of Toronto, Canada

In this article, the authors omitted the ethical approval number and date that approval was obtained from the ethical statement in this publication, which is required under the policies of the journal. The authors confirm that ethical approval was obtained from the Unity Health Toronto Research Ethics Board, for the research. The original approval was granted under REB # 16–375 on February 8, 2017. An extension for biomarker analysis was approved under REB # 20–195 on May 30, 2021.

The original ethics statements found in section 2.1 and the declarations are below:

***“2.1. Ethical approval***

*The Unity Health Toronto research ethics board approved this study. All patients provided informed consent and the study was performed according to principles outlined in the Declaration of Helsinki [16].”*

***“Ethics statement***

*This study was granted approval by the Unity Health Toronto Research Ethics Board (UHT-REB). All patients provided informed consent and study procedures were conducted according to the principles outlined in the Declaration of Helsinki.”*

Both statements have been updated to include the original and extension grant numbers and dates.

The correct version of the ethics statements found in section 2.1 and the declarations should be as below:

***“2.1. Ethical approval***

*The Unity Health Toronto research ethics board approved this study.****The original approval was*** granted ***under REB # 16–375 on February 8, 2017. An extension for biomarker analysis was approved under REB # 20–195 on May 30, 2021.*** *All patients provided informed consent and the study was performed according to principles outlined in the Declaration of Helsinki [16].”*

***“Ethics statement***

*This study was* granted *approval by the Unity Health Toronto Research Ethics Board (UHT-REB).* ***The original approval was*** granted ***under REB # 16–375 on February 8, 2017. An extension for biomarker analysis was approved under REB # 20–195 on May 30, 2021.*** *All patients provided informed consent and study procedures were conducted according to the principles outlined in the Declaration of Helsinki***.***”*

## Declaration of competing interest

The authors declare that they have no known competing financial interests or personal relationships that could have appeared to influence the work reported in this paper.

