# Supplementary material for: Angiogenesis-related proteins as biomarkers for peripheral artery disease
Source: Heliyon. 2023 Sep 15;9(9):e20166. doi: 10.1016/j.heliyon.2023.e20166 (PMC10559913; doi:10.1016/j.heliyon.2023.e20166)
Supplement: Multimedia component 1 [file mmc1.pdf]

**SUPPLEMENT**

**Supplemental Table 1: Three-year adverse peripheral artery disease related event rates stratified by Fontaine classification**

| | **Non-PAD (n=125)** | **I (n=41)** | **IIa (n=54)** | **IIb (n=125)** | **III (n=30)** | **p-value** |
|---|---|---|---|---|---|---|
| Major adverse limb event (MALE) | 0 (0) | 0 (0) | 17 (31) | 38 (30) | 28 (93) | **<0.001** |
| Vascular intervention | 0 (0) | 0 (0) | 17 (31) | 36 (29) | 26 (87) | **<0.001** |
| Major amputation | 0 (0) | 0 (0) | 2 (4) | 6 (5) | 9 (30) | **<0.001** |
| Worsening PAD status | 0 (0) | 0 (0) | 25 (46) | 21 (17) | 6 (20) | **<0.001** |

Results presented as N (%).
All p-values were rounded to three decimal places, with statistical significance set at two-tailed P < 0.05 (bold).
Abbreviations: PAD (peripheral artery disease).

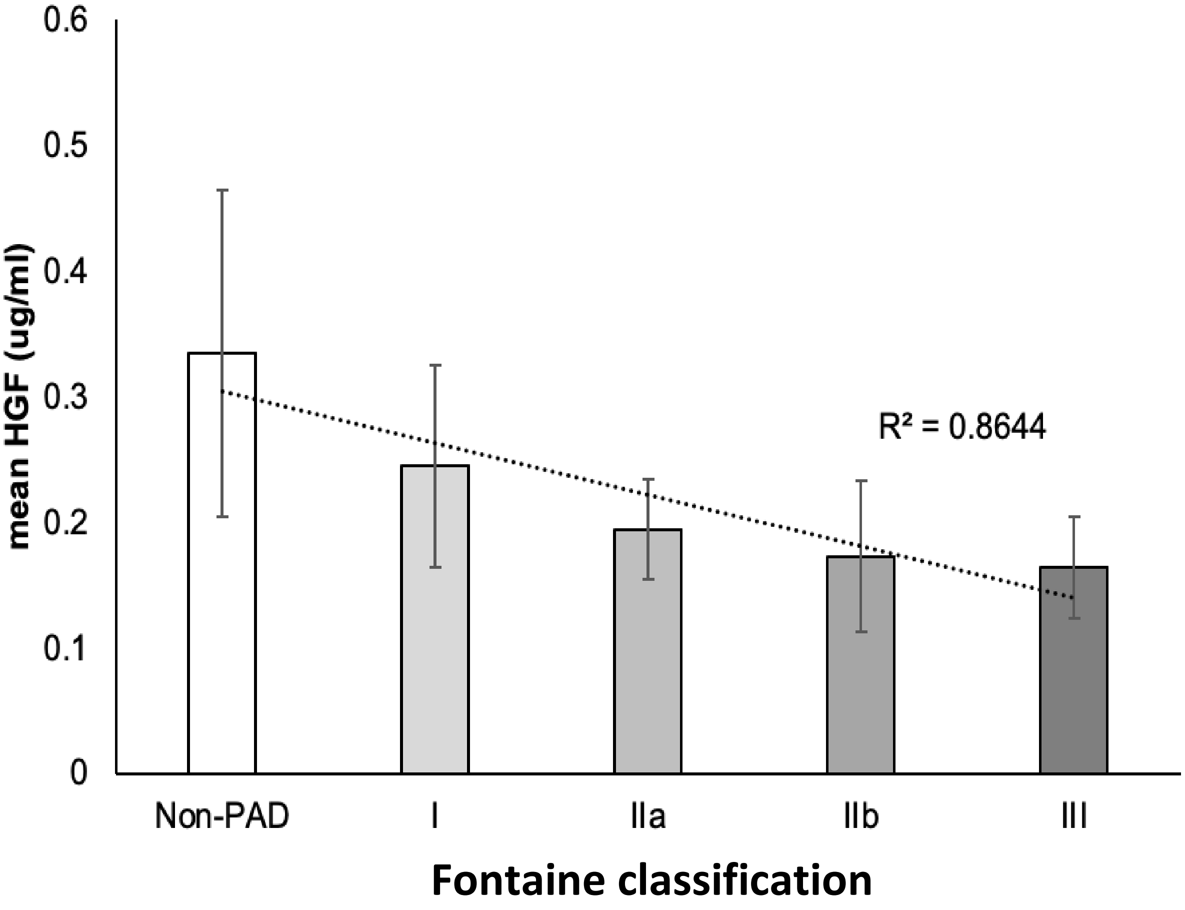


**Supplemental Figure 1: Hepatocyte growth factor (HGF) levels based on Fontaine classification of peripheral artery disease (PAD).** Error bars represent standard deviation. Dotted line represents the trend, measured using the coefficient of determination ($R^2$).
